# The shifting culture of the scientific workforce – a change for women and girls in science

**DOI:** 10.1242/dmm.050111

**Published:** 2023-02-09

**Authors:** Kirsten C. Sadler

**Affiliations:** NYU Abu Dhabi, PO Box 129188, Abu Dhabi, UAE

## Abstract

Celebrating, educating and mobilizing the global community to achieve equality for women and girls in science is recognized worldwide each February 11 on the International Day of Women and Girls in Science. This day serves as a rousing call for people of all genders to gain unfettered access to opportunities to become successful, engaged and productive scientists. One strategy to achieve this is to develop policies to ensure that all people can pursue science education, training and careers, unimpeded by their sex, gender and gender identity. Another strategy is to assure representation of women and non-binary people at every level of the scientific workforce. Collectively, these strategies and other important efforts are shifting the global mindset to one where women and girls are recognized as vital, capable and innovative contributors to disciplines that have historically been dominated by men. These male-dominated disciplines have evolved cultural norms rooted in masculine stereotypes and the next challenge is to continue this shift away from a scientific culture in which hypercompetitive, individualistic, solo artists work alone in single-minded pursuit of a breakthrough. Instead, the reality is that contemporary biomedical scientific research is a cosmopolitan culture; one where breakthroughs depend on collaboration, where data sharing is the norm and where all are encouraged to contribute their best ideas to help solve science's most vexing and exciting problems.

## Teaser

**Summary:** The International Day of Women and Girls in Science is a celebration of the achievements of female scientists, and a call to action for a culture shift in science.

## Global celebration of women and girls in science

The United Nations (UN) work to address issues that affect us all. These issues are highlighted by International Days, which are designated as “occasions to educate the public on issues of concern, to mobilize political will and resources to address global problems, and to celebrate and reinforce achievements of humanity”. One issue of concern worldwide is that women remain underrepresented in the scientific workforce. Therefore, in 2015, the UN declared February 11 to be the International Day of Women and Girls in Science (IDWGS). On this day, celebrations, symposia, conferences, parties and social media campaigns occur simultaneously across the globe as a unified recognition of the contributions that women and girls have made, and are making, in science, and also to draw our focus to the remaining barriers.

The IDWGS was launched the same year I transitioned from New York City to the United Arab Emirates to assume a new position – first as biology professor now as Vice Provost – at New York University Abu Dhabi (NYUAD). I have celebrated the IDWGS each year in what is now my home. We plan this annual event in collaboration with fellow faculty, students and researchers, who come to Abu Dhabi from across the world and, through this, I have learned that, while some of the barriers facing female scientists are universal, others are culturally distinct. In 2022, over 30 countries had no laws prohibiting discrimination in employment on the basis of gender. In 2018, over 100 countries had restrictions in the types of job women can hold – with most restrictions related to work activities deemed dangerous (World Bank, 2022). Added to this are societal expectations that discourage girls from pursuing careers in areas like science, engineering and technology that have been traditionally male dominated. The UN are addressing these issues directly through their Sustainable Development Goals 4 and 5 that aim to ‘ensure inclusive and equitable quality education, and promote lifelong learning opportunities for all’ and ‘achieve gender equality and empower all women and girls’ (United Nations, 2022). However, in other areas of the world, the majority of science majors are women, and women are entering and rising in the scientific workforce at an unprecedented pace. Examples include Jordan, where 60% of all natural science graduates are women; Georgia, where nearly half the workforce in science, technology, engineering and mathematics (STEM) are women; and the UAE, where women comprise 56% of graduates in STEM courses at government universities and where the Gender Balance Council is hard at work to assure “equity […] and balance in decision-making positions” across sectors. The percentage of biology bachelor's degrees awarded to women in the US in 2014 was 59.1% (National Center for Science and Engineering Statistics, 2021), which was followed by encouraging data, showing that, in 2019, women made up 48% of the US life sciences workforce (Pew Research Center, 2021).

Despite these advances, the percentage of women in the global scientific workforce has stubbornly hovered around 30% for over a decade (UNESCO Institute for Statistics, 2019). Furthermore, the proportion of women in senior leadership roles remains low, with under 20% of STEM professors in the US in 2015 being women (Association for Women in Science, 2019). This is attributed, in part, to girls and women being both deterred from entering science and, also, to their leaving science, even after pursuing an advanced degree. One study examining publishing careers across the globe for more than 60 years – up to 2010 – provided the hard numbers to support what many of us active in the workforce have seen first-hand: female scientists leave academia at a much higher rate than their male counterparts (Huang et al., 2020). Although there are promising signs of a shifting pattern in academic hiring in the US (Williams and Ceci, 2015), the STEM gender gap persists across most of the world.

The efforts to get women, girls and non-binary people to walk through the door of the lab should be matched by efforts to get them to stay, contribute and thrive. Recent analysis showed that a high rate of women are leaving science because of persisting gender pay gaps (Woolston, 2021), bias and exclusion (Puritty et al., 2017), as well as the pressures of parenthood (Cech and Blair-Loy, 2019). Crucial to turning the tide is promoting a culture in science that welcomes participation of people of all genders.

My education in the North American system was one that encouraged creativity and curiosity but was overshadowed by the stereotype of science as a field that only welcomes the brilliant, the dedicated, and those who single mindedly pursue their discipline (Allen et al., 2021; Brown et al., 2018; Vial et al., 2022). Through my experience working as a scientist in three countries with students and colleagues who have diverse identities, I have learned that this is not a universal opinion. I now appreciate that the culture of biomedical science is perceived by some to be welcoming, where women can – and do – make meaningful contributions that are recognized to be of equal importance as those from colleagues who are men. This year, as we celebrate the IDWGS, we call for both sharing the good news that there is a culture shift underway in science globally as well as recognizing the importance of overcomeing barriers to this shift in our march towards the UN goal of “Recognizing the role of women and girls in science, not only as beneficiaries, but also as agents of change”.

## Out with individualism – in with communal goals

The goal of gender equity in science extends beyond equal access and opportunity for all genders – it should encourage everyone to engage with science education and pursue careers as scientists. Studies have shown that many girls, women and non-binary students veer away from career tracks that are often perceived as individualistic, isolated and unlikely to advance communal goals of helping others (Allen et al., 2021; Diekman and Benson-Greenwald, 2018). Stereotypical societal feminine norms steer women and girls to areas where they work collectively and collaboratively, devoting their talents to areas with an impact on society. Biomedical science is just the place where this can happen and making this clear to girls as they consider a career path is important. Many discoveries in biology influence treatments for disease, improve health outcomes and mitigate some of the biggest threats of our time, such as food insecurity and climate change. Indeed, as the complexity of biological problems requires diverse teams to tackle them, persisting with a scientific workforce that either explicitly or inadvertently discourages participation of all genders will hinder progress.

Until the middle to end of the last century, medicine and biomedical science research was male dominated. Excluding women and girls from science contributed to the development of cultural norms associated with a male context: of hyper-competition and individualism. This is reflected in the awarding of high-profile prizes to a small number of individuals – even when teams of researchers worked collectively towards the groundbreaking discovery. It is still amplified in the media, where scientists are often portrayed in a poorly lit lab, working alone, late into the night (see image, left). The  ‘bro culture’ – exemplified in Silicon Valley or, its parallel ‘Old Boys Club’ – is one where all-nighters are a norm, admitting mistakes is akin to a weakness and winning is the ultimate goal. This has become the predominant cultural paradigm in some labs, elite graduate programs and research institutes. While this workplace culture may appeal to some, it is off-putting for many girls, women, non-binary people and, indeed, many men, who do not thrive in overly competitive and individualistic workplaces.

**Figure:**
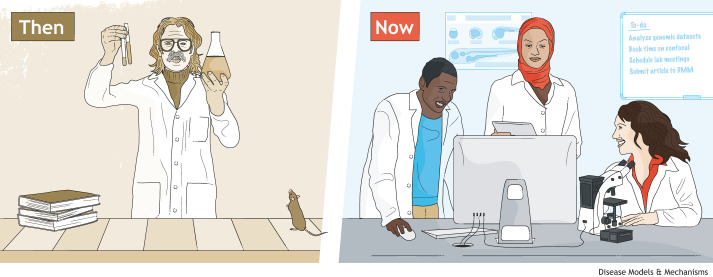
This image is by neilsmithillustration.co.uk and published under our CC-BY license.

Contrast this to the collaborative culture I experience as a professor and member of academic leadership at NYUAD, as well as the experiences shared by undergraduate students I interviewed for this article. We find that *how* we work is just as important as *what* we work on and the skills that we each bring to a project. When I started on my career path as a biologist, I was excited to pursue all opportunities offered to me. In the process of doing so, I set aside personal interests and immersed myself in the projects I was working on while forming bonds with my lab members as we spent hours together working at the bench, discussing science, politics, music and life. Experimental science was more social than I ever thought it could be: my peers were friendly, helpful and interesting. Many have remained close friends and colleagues. As I formed my own lab, I worked to cultivate a culture where preliminary data are shared openly and where mistakes are accepted – even celebrated as a learning opportunity. I work hard to build a foundation of respect among all lab members, showing that I am not afraid to be wrong, admit I don't know something or that my hypotheses are not supported by the data. I am rewarded by seeing that members of my lab have grown deep connections while sitting side by side at the microscope for hours every day, and they have established ways of working so that the lab benefits collectively. Our ‘all hands-on-deck’ policy when a paper is about to be submitted means that everyone who is engaged with a project pitches in to help get it ready for publication and, in this fashion, the success of one benefits all. While I and most members of my team find this culture does work to bring out our best, the engines that generate promotions, awards and recognition in science have not adjusted to this way of collaborative working, and more-individual achievements are still used as a basis for career advancement.

Biology is inherently social and, if science is continually perceived as being disconnected from communal goals and helping others – which Western society has traditionally ascribed as feminine traits – achieving gender equity in science will continue to meet roadblocks. Instead, I propose an advertising campaign, where we portray the contemporary reality of a career in life sciences – one that is highly social, intensely collaborative and focused on achieving communal goals, towards which scientists work together. This is already a reality in many scientific workplaces across the world and, as we celebrate the IDWGS, we are all called to promote this ethos so we can deliver this promise.

## It's not (only) about numbers

Equity is often about numbers, with a goal of achieving a proportion of men, women and non-binary people pursuing a career in science that reflects the world population. Efforts across the global scientific workforce – including the IDWGS – are focused on getting women through the door of the lab or hired into faculty ranks. As we move towards equity, we need to embrace different perspectives and experiences, as these are essential to solving complex scientific problems. Creating an inclusive environment, minimizing bias and maximizing outreach to women are all standard practice in hiring. However, as one recent biology graduate shared in a focus group I ran as part of the research for this article “representation and numbers are important, but […] if you hire equal numbers of men and women, everyone could be very competitive – and then it wouldn't make science more inclusive […] It is just a false representation”. I agree. Essentially, we need to ensure that we are not only hiring women who ascribe to the stereotypical masculine cultural norms, but support women and men who have approaches to science, leadership, communication, mentoring and teaching that deviate from the traditional ideals defined by masculine traits. Recruiting women who can be agents of change in the culture of science would shift us away from ‘bro culture’ to ‘co(llaborative) culture’, where everyone can apply their skills, talents and ideas to the scientific workforce.

## Leadership matters

Several years ago, when I asked my mentor Nancy Hopkins what was the key to advancing equity in all her years of experience working on gender equity in science, she simply replied “it comes from the top”. In short, leadership matters. It is the leadership of universities, industries, institutes and hospitals that make policies, set the culture and create paths to success. It is these leaders who can make real change shifting to ‘co-culture’. The percentage of women in scientific leadership is vanishingly small worldwide (Association for Women in Science, 2019; European Commission, 2019), and of those who have assumed these positions, many report needing to distance themselves from stereotypically feminine traits in order to be successful. Leaders of all genders who embrace collaboration, celebrate success of others and can embrace the opportunity to learn from mistakes are those who will create a workplace where talent flocks. A true test of whether this culture has shifted in academia will be when leaders recognize that collaborative science is a sign of success, so that those who work together are those who are promoted.

In virtually every area, women are held to a double standard as leaders – where being caring can be interpreted as soft, where contemplation can be interpreted as indecision and, by contrast, decisiveness, persistence, critical thinking and striving for excellence, can be interpreted as harsh, insensitive or bossy. We need to embrace leadership styles based on personal attributes, not gender-based expectations. As we celebrate the achievements of female scientists, let's also invite more women and men who hold these values to take the helm in steering scientific agendas, and leading our universities and organizations.

## Real choice

The idea behind the IDWGS is to achieve real equity in science, so that women, girls and non-binary people with a passion for scientific discovery can proceed with fervor towards that goal. Put otherwise, equity means that the choice to become a scientist will be based on interests and capacity – not on gender. In the decades since I started my career as a scientist, I have seen this culture shift towards one where a girl declaring that she wants to be a biologist is no longer met with raised eyebrows but with a nod and a smile. I have seen graduate programs transform from a funnel through which only the most brilliant are seen to pass, into what is a true essence of advanced study: a forum for training students to do good and impactful science. In the next decades, I hope to see that the perceptions of science shift away from the stereotypical nerdy, lone genius, toiling late into the night to crack some difficult code to one where hard-working and dedicated people of all genders bring their best ideas, share their data and tackle the issues the world needs solved.
